# Guiding Breathing at the Resonance Frequency with Haptic Sensors Potentiates Cardiac Coherence

**DOI:** 10.3390/s23094494

**Published:** 2023-05-05

**Authors:** Pierre Bouny, Laurent M. Arsac, Antoine Guérin, Guillam Nerincx, Veronique Deschodt-Arsac

**Affiliations:** 1 Univ. Bordeaux, CNRS, Bordeaux INP, IMS, UMR 5218, F-33400 Talence, France; pierre.bouny@u-bordeaux.fr (P.B.); veronique.arsac@u-bordeaux.fr (V.D.-A.); 2 URGOTECH, 15 Avenue d’Iéna, F-75116 Paris, France; aguerin@urgotech.fr

**Keywords:** cardiac coherence, resonance frequency breathing, haptic sensor, HRV biofeedback, anxiety, stress

## Abstract

Cardiac coherence is a state achieved when one controls their breathing rate during the so-called resonance frequency breathing. This maneuver allows respiratory-driven vagal modulations of the heart rate to superimpose with sympathetic modulations occurring at 0.1 Hz, thereby maximizing autonomous power in heart-to-brain connections. These stimulations have been shown to improve vagal regulations, which results in obvious benefits for both mental and organic health. Here, we present a device that is able to deliver visual and haptic cues, as well as HRV biofeedback information to guide the user in maintaining a 0.1 Hz breathing frequency. We explored the effectiveness of cardiac coherence in three guidance conditions: visual, haptic and visuo-haptic breathing. Thirty-two healthy students (sixteen males) were divided into three groups that experienced five minutes of either visual, haptic and visuo-haptic guided breathing at 0.1 Hz. The effects of guidance on the (adequate) breathing pattern and heart rate variability (HRV) were analyzed. The interest of introducing haptic breathing to achieve cardiac coherence was shown in the haptic and visuo-haptic groups. Especially, the *P*_0.1_ index, which indicates how the autonomous power is ‘concentrated’ at 0.1 Hz in the PSD spectrum, demonstrated the superiority of combining haptic with visual sensory inputs in potentiating cardiac coherence (0.55 ± 0.20 for visuo-haptic vs. 0.28 ± 0.14 for visual only guidance; *p* < 0.05) haptic-induced effectiveness could be an asset for a more efficient and time-saving practice, allowing improved health and well-being even under tight time constraints.

## 1. Introduction

Cardiac coherence describes a state where heart rate variability (HRV) exhibits a unique oscillatory frequency with large oscillations [1,2,3,4]. Achieving cardiac coherence improves bottom-up vagal stimulations of the brain [5,6,7]. Although a number of methods exist that can be used to achieve cardiac coherence, it has been stressed that maintaining a precise breathing frequency may achieve maximal benefits. This line of thought promotes the development of accurate devices to enhance the best practice of guided breathing. Here, we were interested in the role played by haptic guidance when it was combined with (more classical) visual biofeedback in order to stimulate adequate breathing behavior and potentiate cardiac coherence.

Reaching perfect cardiac coherence and maintaining it for minutes is not trivial. A number of experimental studies have shown obvious benefits for health and well-being after a few sequences [5,6,8,9,10,11] or even one sequence of cardiac coherence practice [12,13]. The benefits are attributed to the exacerbated power in heart-to-brain bottom-up connections during practice, which is a condition that drives brain network remodeling. Improved functioning of the central network processes has critical effects on perceptual, cognitive and emotional regulation in patients, which are described, e.g., in terms of improved stress management, decreased PTSD symptoms and better attentional skills [14,15]. In healthy people, the practice helped them to perform better in cognitive tasks and improved the management of stressful situations [12,16], even among highly trained athletes engaged in important competitions [17].

The heart–brain interplay has been described through a functional network [18,19,20] linking the central nervous system (CNS) and the autonomous nervous system (ANS). Assessing the particular functioning of the so-called Central Autonomic Network can help researchers to grasp the main (dys)functions in both mental [21,22] and organic health [19,23]. It is known for years that the heart continuously sends bottom-up stimuli to the brain [24]. Specific bottom-up stimulation sent through the heart-to-brain network can have significant benefits for brain functions [23,24,25]. Observing obvious benefits requires that the power in HRV-driven afferences is maximized for minutes, which is a typical behavior associated with cardiac coherence. The control of breathing to achieve so-called resonance frequency breathing is an efficient way to achieve cardiac coherence.

The short-range regulation of the heart rate by sympathetic and vagal branches of the autonomous nervous system (ANS) can be conceived as a two-oscillator system, affecting the basic heart rhythm imposed by the sinus node. The dominant influence on short-term HRV of the sympathetic drive results in a 0.1 Hz rhythm emerging from a central descending command and the baroreflex loop linked to vasomotor control of whole-body vascular beds (Mayer waves). Concurrently, modulation by the parasympathetic system is driven by the spontaneous 0.2–0.3 Hz breathing rate, which is reflected in similar 0.2–0.3 Hz oscillations in heart rate variability (HRV), known as Respiratory Sinus Arrhythmia (RSA). When one voluntary reduces their breathing rate to a rhythm approaching 0.1 Hz, these two oscillators are in resonance. This specific state is identified as cardiac coherence; HRV exhibits a smooth quasi-sinusoidal signal output at around 0.1 Hz with maximal magnitude, which is the desired effect. The best practice of cardiac coherence needs, therefore, one’s breathing rate to be guided, which could be associated with feedback information about the HRV sinusoidal signal to confirm perfect achievement. This so-called HRV biofeedback has gained significant interest [1,3,4].

In the present study, we present a specific device that is able to deliver visual, haptic and visuo-haptic breathing guidance, as well as HRV biofeedback. The main objective was to guide the breathing rate in order to generate cardiac coherence and maintain this particular state for several minutes. We hypothesized that combining visual and haptic sensory inputs would allow the achievement of cardiac coherence through a tight control of the 0.1 Hz-guided breathing rate.

## 2. Methods

### 2.1. Participants

A total of 32 students of the faculty of sport sciences gave their informed consent to participate to this study, which was part of their academic curriculum and for which they received credits. The IRB of the ‘faculté des STAPS’ endorsed all the procedures, which followed the rules of the Declaration of Helsinki and its later amendments. The participants were randomly assigned into three experimental groups, each performing a specific guided breathing modality: visual or haptic or visuo-haptic (Table 1).

All these young and healthy sport students were free of prior cardiovascular disease, severe inflammation, psychological disorders and did not take (cardiovascular) medication. They had no experience of cardiac coherence practices. Consuming alcohol and caffeine and performing heavy physical activity were prohibited during the 24 h preceding the experiment.

### 2.2. Protocol

**Figure 1:**
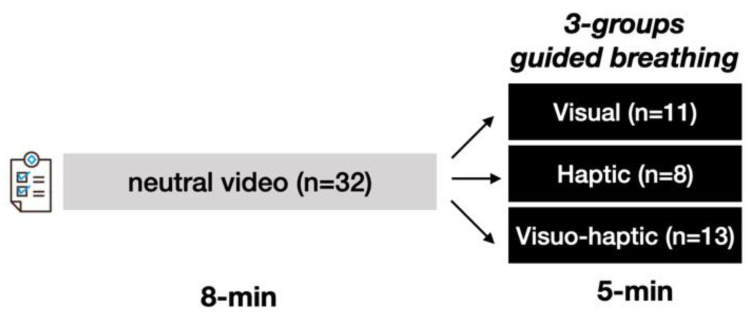
The experimental protocol began with filling questionnaires, and then watched an emotionally neutral video for 8 min, breathing at a spontaneous rate. After a short pause, all participants received breathing guidance for 5 min, accompanied by visual HRV biofeedback, except in the haptic group, where visual guidance was not given. HRV and breathing rate were monitored during the whole protocol.

Upon their arrival at the laboratory, the participants filled out questionnaires and were equipped with chest electrodes for ECG recording. Then, they remained seated and relaxed, facing a 15.6″ screen of a computer on a table (viewing distance ± 0.5 m). For each experimental group, the main sequence consisted of 5 min of guided breathing at 0.1 Hz using a specific modality: visual, haptic or visuo-haptic. This sequence was preceded by the viewing of an emotionally neutral video during which the participants breathed at spontaneous rate. The protocol is illustrated in Figure 1.

### 2.3. The Device

**Figure 2:**
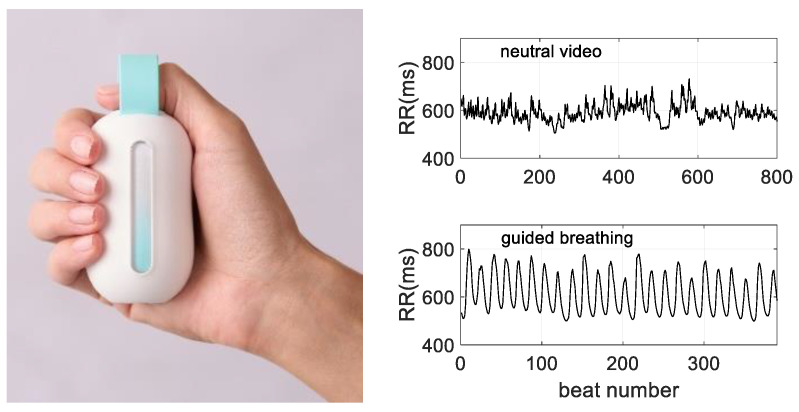
**Left** panel: The device used for guiding breathing and delivering HRV biofeedback. For visual guidance, the light goes up and down in 5 s phases to guide inspiration-expiration cycles at the resonance frequency breathing (0.1 Hz). For haptic guidance, vibration intensity increases and decreases following the same pattern. When the visual was given, HRV biofeedback was delivered thanks to fading from blue to orange when HRV was not smoothed (see method). **Right** panel: A typical HRV measures during viewing the video (top) and performing visuo-haptic respiratory guidance (bottom).

Guided breathing to achieve the resonance frequency was achieved by holding a specifically designed device in the dominant hand (Figure 2). The device paced a breathing rate of 0.1 Hz by providing 5 s inspiration and 5 s expiration timed sequences along with visual, haptic or visuo-haptic guidance. The haptic stimulation consisted of vibrations, whose frequency increased for 5 s, and then decreased for 5 s. When visual cues operated (visual and visuo-haptic conditions), HRV biofeedback was provided thanks to color changes on the device. For this, the light seen in the device window (Figure 2) faded from blue to orange when the recorded HRV did not show a smooth rhythm. The smoothness of HRV was detected with a photoplethysmography (PPG) sensor placed below the thumb of the user, which detects blood wave pulses as a surrogate for heart rate. The pattern was compared to the 0.1 Hz expected sine wave with the verification of the monotonic nature of the HR fluctuation over 5 s phases (i.e., continuous increase during inspiration and continuous decrease during expiration).

### 2.4. Measurement of Heart Rate Variability

The heart rate was obtained thanks to a bipolar electrodes system connected to a PowerLab 26T (ADInstruments, Dunedin, New Zealand), which was used to perform sampling at 1 kHz. From the QRS complex of this 2-lead ECG, Rpeak-to-Rpeak detection was performed in Matlab (Matlab Release 2021a, Mathworks, Natick, MA, USA) using custom routines. Heart rate variability (HRV) was explored in the form of RR series inspected for artifacts, occasional ectopic beats (irregularity of the heart rhythm involving extra or skipped heartbeats, e.g., extrasystoles and consecutive compensatory pauses). When necessary, ectopic and artifact data were manually replaced with interpolated adjacent values. Less than 2% of the total samples were corrupted in the analyzed series.

### 2.5. Measurement of the Breathing Rate

The breathing rate was obtained in synch (1 kHz) with the ECG signal thanks to a respiratory belt connected to another channel of the same PowerLab device. Breathing time series were imported into Matlab for subsequent analyses using both Matlab existing functions and custom-designed algorithms.

### 2.6. Analyses of HRV Dynamics

In order to quantitatively describe how the guidance helped the participant to achieve cardiac coherence, we calculated three main estimators of HRV in time and frequency domains.

In the time domain, the successive peak-to-peak intervals duration in HRV were computed. Then, they were compared to a perfect series of 10 s interval durations by calculating the root-mean-square error (RMSError) as:

(1) $$\mathrm{RMSError}=\sqrt{\frac{1}{N}\sum_{i=n}^{N}|x_{n}-10{|}^{2}}\;$$

where $x_{n}$ indicates the cardiac cycle duration in seconds, and N is the number of cycles in the series.

In the frequency domain, the RR time series was analyzed using Fourier Transform after 4 Hz resampling using a cubic spline interpolation. Any trend as well as the very low frequencies in HRV were removed by using a filtering procedure specifically developed for HRV analysis [26] using lambda = 300, which corresponds to a cutting frequency of 0.042 Hz.

The power spectral density (PSD) of the HRV was used to verify that maximal power in HRV was observed at 0.1 Hz during guided breathing.

To provide a third estimator, the PSD obtained during video viewing was also used comparatively with the PSD during guided breathing. The main intuition was to quantify how autonomic power that is distributed across a range of HRV frequencies (0.04–0.4 Hz) during spontaneous breathing actually concentrated at the expected frequency of 0.1 Hz during guided breathing. For each participant, the index called *P*_0.1_ was thus obtained by dividing the peak height of the PSD during guided breathing by calculating the cumulative sum of peaks heights in the range 0.04–0.4 Hz in the PSD of the video sequence.

(2) $$P_{0.1}=Hmax_{guide}/\sum_{f=0.04}^{f=0.4}H_{video}$$

Clearly, a value of *P*_0.1_ close to 1 means that HRV oscillations that were distributed along the frequency range 0.04 Hz–0.4 Hz during spontaneous breathing are accumulated at the guided frequency 0.1 Hz during cardiac coherence breathing.

### 2.7. Analyses of the Breathing Rate

Similar analyses were performed on the respiratory signal, except for *P*_0.1_. Peak-to-peak series were obtained from the 1 Hz resampled signal obtained with the chest belt, and the RMSError was calculated using breathing cycles, such as in Equation (1). Breathing cycles were determined as the peak-to-peak delay in respiratory signal (i.e., end of each inspiration). Individual PSDs were obtained to confirm the 0.1 Hz respiratory rate. In contrast with the HRV analysis, it must be said that some participants (N=11) were discarded from the respiratory analysis due to a lack of a sufficient signal-to-noise ratio in the signal obtained with the chest belt in our conditions.

### 2.8. Statistical Analysis

Values are expressed as mean and standard deviation (SD). Outliers were identified and corrected using the interquartile range method described by Tukey [27]. The prerequisites for parametric tests were checked using the Shapiro–Wilk test (normality) and Levene test (homogeneity of variance in samples).

To test the effects of the guided breathing modality on cardiac and respiratory parameters, ANOVA, in a 3 × 1 design (group between-factor (visual, haptic, visuo-haptic), was used.

For post hoc tests, we applied the Holm’s correction for multiple comparisons to neutralize to the potential increase in Type I errors.

In addition, Bayesian tests (log(BF10), see Bouny et al. [28] for an interpretation scale of this parameter) were used to extend insight and guide the interpretation of significance (*p* values), according to the likelihood of the alternative hypothesis versus the null hypothesis [29,30].

Data were analyzed in JASP (version 0.17.1.0, https://jasp-stats.org (accessed on 12 April 2023)) and R (version 4.2.0, R: The R Project for Statistical Computing (https://r-project.org (accessed on 12 April 2023))).

In the main figures, results were produced using the NotBoxPlot function (1.31.0.0 https://github.com/raacampbell/notBoxPlot (accessed on 27 November 2022)) developed in MatLab. These figures show the mean (red line), 1 SD (shaded area) and the 95% confidence interval for the mean of each group (light shaded area). The individual values are also overlaid.

## 3. Results

### 3.1. HRV Dynamics during Guided Breathing

**Figure 3:**
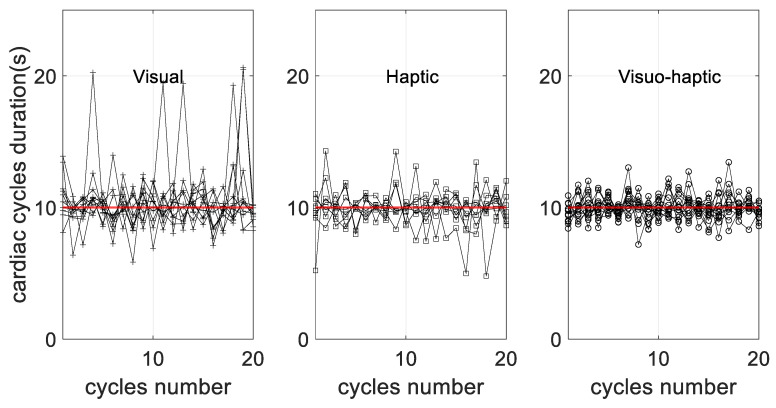
Duration of successive cardiac cycles along the 5 min guided breathing sequence in each guidance condition. The red line indicates the target duration provided by the device, 10 s, so that deviation from guidance could be visualized.

**Figure 4:**
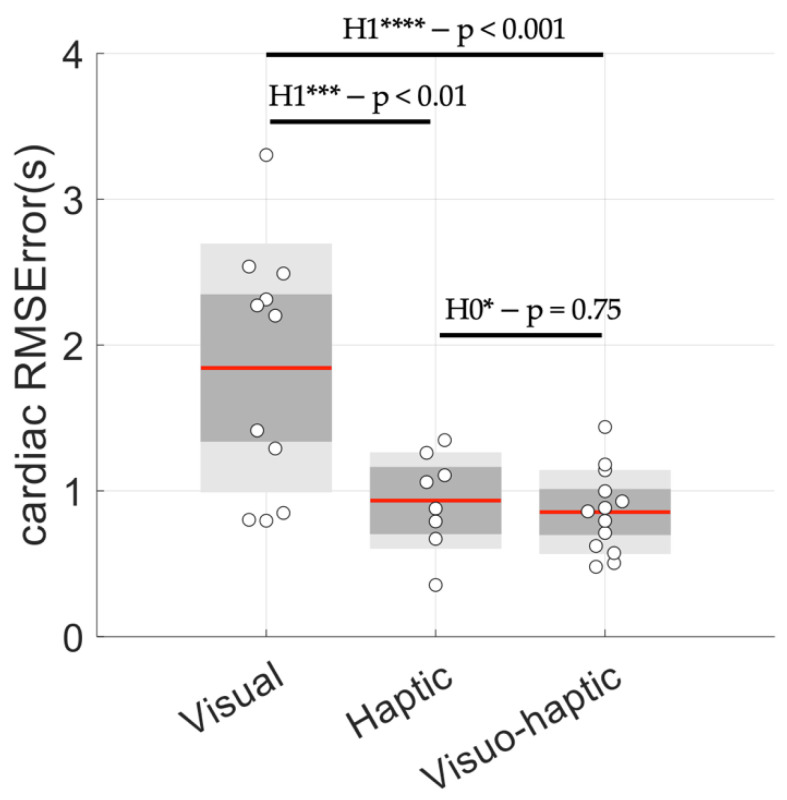
Root-mean-square error obtained from values illustrated in Figure 3. Evidence for the alternative hypothesis (H1**** extreme; H1*** very strong) and for the null hypothesis (H0* moderate). Red line: mean, grey: ±1 SD; light grey: 95% CI.

Peak-to-peak interval durations in the oscillations of the cardiac cycles are illustrated in Figure 3 for 20 successive cycles, excluding the first five and the last five cycles (a total of 30 cycles were obtained at 0.1 Hz for 5 min). They can be visually compared to a perfect 0.1 Hz (10 s) behavior, as illustrated by the red line. The main observations rely on the absence of a trend in terms of successive errors, as well as a minimal error when haptic stimulation was used (respectively, 1.84 ± 0.85 s for the visual group, 0.93 ± 0.33 s for the haptic group and 0.86 ± 0.29 s for the visuo-haptic group), which was quantified with the RMSError obtained for each group (Figure 4).

### 3.2. Power Spectral Density

**Figure 5:**
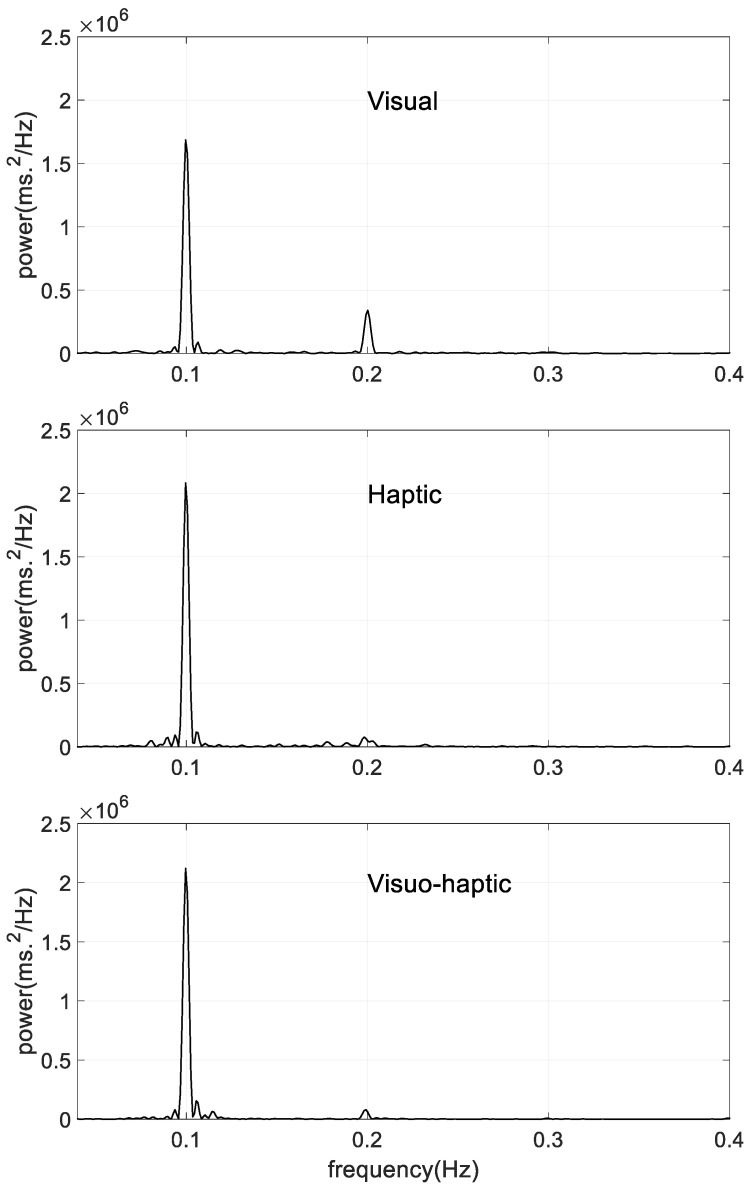
Typical PSDs obtained during guided breathing phase from the HRV analysis in a representative individual of each subgroup.

In good agreement with the time domain analysis of HRV, the Power Spectral Density showed clear peaks in the 0.1 Hz area, as illustrated by individual examples in Figure 5. To quantify this observation, three main characteristics of the peak are compared in Table 2: peak location, peak height and peak width at half-height.

### 3.3. Cardiac Coherence Assessed by P_0.1_

**Figure 6:**
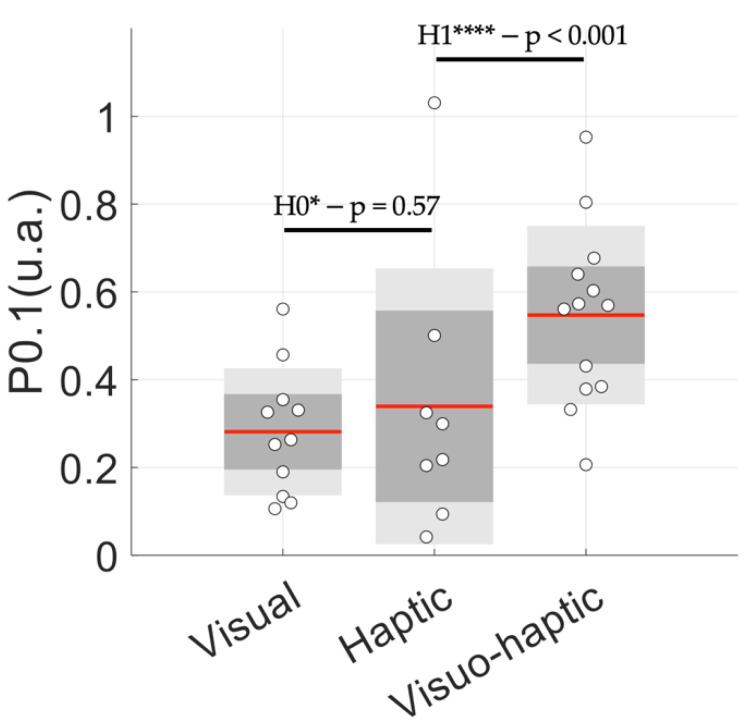
Relative power under the resonance peak at 0.1 Hz (named *P*_0.1_) for each subgroup, illustrating how HRV oscillations distributed in the frequency range 0.04–0.4 Hz during spontaneous breathing is accumulated at 0.1 Hz during cardiac resonance breathing. H1**** and H0* mean, respectively, extreme evidence for the alternative hypothesis and moderate evidence for the null hypothesis. Red line: mean, grey: ±1 SD; light grey: 95% CI.

The analysis of the *P*_0.1_ index showed that cardiac coherence was better achieved in the visuo-haptic group (Figure 6). Remarkably, in this group, the *P*_0.1_ was two-fold higher than it was in the visual group (0.55 ± 0.20 vs. 0.28 ± 0.14).

As an additional finding, Figure 6 shows a higher inter-individual variability in the haptic group (0.34 ± 0.31) compared to those of the other guidance modalities, indicating that the effectiveness of haptic guidance could potentially be more dependent on an individual’s sensitivity to haptic perception.

### 3.4. Control of Breathing Behavior

As expected, guided breathing provided a series of breathing cycles lasting 10 s, characterized by a small RMSError (Table 3), which is also illustrated by clear 0.1 Hz peaks in the PSD of each group, with no difference in the peak location (Table 3).

## 4. Discussion

Achieving cardiac coherence strengthens the heart–brain interplay through enhanced autonomous power (HRV oscillations), which has been shown to improve emotional and behavioral regulations, and more generally, health and well-being. Researchers have recurrently explored a number of ways to achieve cardiac coherence through resonance frequency breathing [10], including those with a special focus on biofeedback conditions that can be used to create an efficient practice [31]. It has been shown that even simple paced breathing (without biofeedback) may offer sufficient conditions to achieve an increase in HRV and related benefits [32]. The central question we addressed in this study was whether combining haptic breathing with visual cues helps people to achieve resonance frequency breathing, thereby facilitating cardiac coherence. For this, we analyzed the heart rate oscillations during visual, haptic and visuo-haptic guided breathing delivered by a new device/sensor.

Strictly speaking, cardiac coherence is effective as long as resonance frequency breathing can be maintained. In turn, successful breathing is reflected in the dominance of a single 0.1 Hz wave in HRV oscillations. In the past, visual cues have been used as an effective guide. They also served as visual feedback indicating that the heart rhythm has reached a smooth, 0.1 Hz oscillating pattern. Our results confirm the positive role of visual guidance through analyses of HRV patterns during resonance frequency breathing (Figure 3). However, more importantly, we noted that there was a significant added value when haptic sensing was used. As a first observation, when our participants were guided through haptic breathing alone, a situation where visual feedback was absent, they were able to achieve cardiac coherence with comparable ease as they did when visual biofeedback was present (Figure 3). This observation emphasizes the effectiveness of using an haptic sensory input when guiding humans through voluntary control, which has been highlighted in other domains, such motor learning for instance. Yet, it was noticed that using the ‘haptic alone’ modality of the present device introduced greater heterogeneity in the performance of cardiac coherence (Figure 6). Although interpreting this observation is beyond the scope of the present study, it may be that sensory inputs from haptic stimuli may not be perceived with the same acuity by everyone. The manifestation of the individuality of the participants in haptic sensing has been shown in other studies [33,34], where it is underlined that haptic sensing depends on motivation, the focused attention given, and the cognitive and emotional statuses in a task-/subject-dependent manner. Considering that achieving cardiac coherence is of particular interest when one faces a difficult situation, a situation wherein specifically cognitive, motivational and emotional manifestations are challenged, the interplay between haptic stimulations and the gain in HRV oscillations deserves further exploration.

In our conditions, visual guidance showed a relative lack of an effect on breathing rate (RMSError in Table 3). This observation might explain why Tabor et al. [32] showed that biofeedback per se brought about a poor benefit for improving HRV oscillations. Here, by adding haptic cues to visual cues, we showed the clear superiority of visuo-haptic conditions based on lower RMSError (Figure 4), and mainly, our *P*_0.1_ index (Figure 6).

As neurophysiological bases of cardiac coherence are associated with perfectly regular and maximal oscillations in HRV when it is maintained for minutes, it can be concluded that visuo-haptic guidance represents the most efficient practice when it is compared to visual or haptic guidance alone. Why visuo-haptic stimulation has better efficacy may have different, non-exclusive explanations. First, it has been shown that the breathing frequency resonance is not exactly 0.1 Hz for every person [2,3]. The baroreflex loop and central command of the natural frequencies of people may be shifted a bit away from 0.1 Hz. In this vein, recent brain mapping using BOLD techniques indicates that descending neural (central command) and ascending vascular oscillations may drive oscillations in HRV to be slightly above 0.1 Hz (0.1–0.14 Hz) and slightly below 0.1 Hz (0.06–0.1 Hz), respectively [35]. The combination of visual and haptic sensory inputs may more intensively rely on multisensory integration when it is compared to visual or haptic alone processing. The role of the central command may become predominant when multisensory integration is present, reducing the impact of baroreflex on resonance and making cardiac coherence more efficiently achievable through a specific heart–brain interplay. As we had no means to test this assumption in our conditions, an interesting idea could be to test people with more or less baroreflex dominance, reflected in 0.06–0.1 Hz dominant HRV power, when experiencing visual vs. visuo-haptic guided breathing. Given the variability in neurophysiological profiles, both in cardiovascular control and perceptual abilities, it is important to consider that the participants in the present study were all young, healthy and active students, which may represent an obvious limitation. Generalizing the results is hardly achievable without testing other populations.

## 5. Conclusions

In this work, we have demonstrated that adding haptic guidance to HRV biofeedback enhances HRV oscillations, which is known as an interesting prerequisite to gaining the ability to perform better emotional and cognitive behaviors. Although different ways of practicing HRV biofeedback have been reported, the present work has its root in the neurophysiological base of the heart–brain interplay to propose that haptic stimulations, possibly through multisensory integration, may potentiate the size of resonant oscillations and associated vagally mediated responses.

Because resonance frequency breathing is an easy practice and has considerable benefits in terms of health and disease, the gain in efficacy demonstrated here, thanks to visuo-haptic guidance, is an asset for acceptability: those who practice it could undergo even short sessions in a tight schedule and benefit from a significant improvement in their emotional and behavioral regulation.

## Figures and Tables

**Table 1:** Demographic parameters of the participants (mean ± SD).

	Visual (N = 11)	Haptic (N = 8)	Visuo-Haptic (N = 13)
Age (years ± SD)	22 ± 4	21 ± 1	20 ± 2
Female (%)	45	25	69

**Table 2:** Descriptive statistics (mean ± SD) of the characteristics of the maximal peak found in the cardiac PSD during guided breathing for each subgroup. The location of the peak and the corresponding power were computed. The mid-peak height width (MPHW) was also measured as an index of the dispersion around the 0.1 Hz target frequency.

	Visual (N = 11)	Haptic (N = 8)	Visuo-Haptic (N = 13)
Peak location (Hz)	0.100 ± 0.001	0.100 ± 0.001	0.100 ± 0.001
Peak height (ms^2^ × 10^6^)	1.97 ± 1.61	1.60 ± 0.48	2.31 ± 1.27
MPHW (Hz)	0.0120 ± 0.0003	0.0121 ± 0.0002	0.0121 ± 0.0003

**Table 3:** Descriptive statistics (mean ± SD) of the location of the maximal peak found in the respiratory PSD during guided breathing for each subgroup and the RMSError parameter measured as per respiratory signal.

	Visual (N = 8)	Haptic (N = 6)	Visuo-Haptic (N = 7)
Peak location (Hz)	0.100 ± 0.000	0.100 ± 0.000	0.100 ± 0.000
RMSError (s)	0.82 ± 0.19 *	0.34 ± 0.15 ^c^	0.45 ± 0.17 ^c^

* indicates significant difference with (^c^) compared group.

## Data Availability

The dataset generated and analyzed during the current study is available from the corresponding author on a reasonable request.
